# Tracking Control of a Magnetic Shape Memory Actuator Using an Inverse Preisach Model with Modified Fuzzy Sliding Mode Control

**DOI:** 10.3390/s16091368

**Published:** 2016-08-25

**Authors:** Jhih-Hong Lin, Mao-Hsiung Chiang

**Affiliations:** Department of Engineering Science and Ocean Engineering, National Taiwan University, No. 1, Sec. 4, Roosevelt Rd., Taipei 106, Taiwan; d98525020@ntu.edu.tw

**Keywords:** magnetic shape memory alloys, magnetic shape memory actuator, hysteresis compensator, Preisach model, inverse Preisach model, modified fuzzy sliding mode control, tracking control, non-linear control system

## Abstract

Magnetic shape memory (MSM) alloys are a new class of smart materials with extraordinary strains up to 12% and frequencies in the range of 1 to 2 kHz. The MSM actuator is a potential device which can achieve high performance electromagnetic actuation by using the properties of MSM alloys. However, significant non-linear hysteresis behavior is a significant barrier to control the MSM actuator. In this paper, the Preisach model was used, by capturing experiments from different input signals and output responses, to model the hysteresis of MSM actuator, and the inverse Preisach model, as a feedforward control, provided compensational signals to the MSM actuator to linearize the hysteresis non-linearity. The control strategy for path tracking combined the hysteresis compensator and the modified fuzzy sliding mode control (MFSMC) which served as a path controller. Based on the experimental results, it was verified that a tracking error in the order of micrometers was achieved.

## 1. Introduction

Magnetic shape memory (MSM) alloys are a new and important member of the class of smart materials that have been widely studied since 1996 [1,2]. MSM alloys are mainly composed of Ni-Mn-Ga composites, but there are also other alternative composites [3]. MSM alloys have the property called magnetic shape memory effect, which means its twin-variants in the phase martensite could be switched. The twin-variants can be induced to switch via the application of magnetic fields, and its maximum magnetic field induced strain is from 6% to 12%. The strain depends on the material composition and modulation [4,5,6]. Shape memory alloys (SMA) exhibit similar behavior, but create large strains up to 8% by changing their temperature, allowing the alloys to switch between the martensite and austensite phases [7]. Compared to SMAs, MSM alloys have the property of higher frequency response, since SMAs use heat transfer for phase change and MSM alloys inherently take advantage of the change of the twin-variants in the same phase with a much faster response. These properties of larger strain and faster response make MSM alloys potential candidate materials for many different applications.

**Figure 1:**
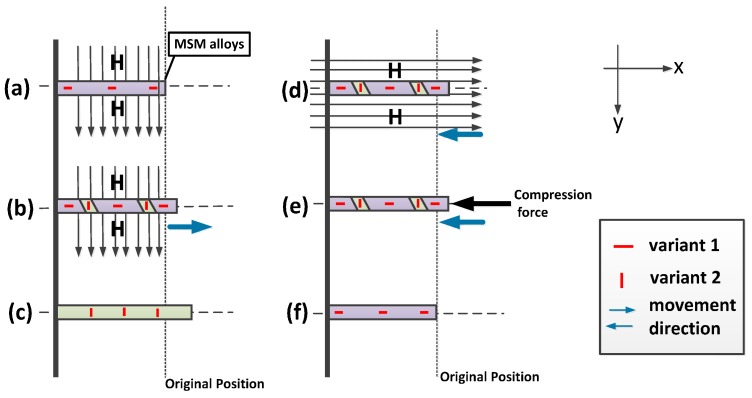
Operational principle of MSM alloys (**a**) MSM alloys have only variant 1 in the first state; (**b**) With the strong magnetic field (>0.7 T) in the *y*-direction, the MSM element elongates in the *x*-direction. Elongation will stop when all the twin-variants switch to variant 2; (**c**) Under small or absence of a magnetic field, MSM alloys can hold their length; (**d**) MSM alloys can contract with the provision of magnetic field in the *x*-direction; (**e**) MSM alloys can contract with the provision of compressive force; (**f**) MSM alloys can return to its original position through process (**d**) or (**e**) [10].

An MSM actuator is an application based on the properties of MSM alloys. Though several composites are found to possess the MSM effect, the material made of Ni-Mn-Ga is chosen here based on its largest magnetic field induced strains within the alternatives [2,3]. In addition, a MSM actuator consists of magnetic circuits, MSM elements and returning force springs so that the function of reversible actuation can be repeated through the elongation and contraction of the MSM elements. The operational principle of MSM alloys is demonstrated in Figure 1 [8]. The strain is due to the switching between the twin-variants of MSM alloys. In the beginning, MSM alloys are maintained in the presence of variant 1, which is shown in Figure 1a. The change from variant 1 to variant 2 is induced when the magnetic field passes perpendicularly through the MSM alloys as shown in Figure 1b. The stronger the magnetic field, the faster the variant 1 can transform to variant 2. The maximum strain is achieved if variant 1 has fully changed to variant 2, as seen in the saturation in Figure 1c. To contract the MSM alloys, which means to reverse variant 2 to variant 1, there are two ways to achieve this goal. One is to use another direction of the magnetic field, perpendicular to the original, as shown in Figure 1d. The other is to add a compressive force to the MSM alloys by inserting a mechanical spring, as shown in Figure 1e. In Figure 1f, the MSM alloys contract to their original position if the magnetic field or the compressive force is strong enough [9].

Since the MSM actuator uses the properties of MSM alloys, it also inherits the drawbacks of MSM alloys, namely the hysteresis non-linearity caused by the transformation of twin-variants and temperature changes [11]. Many researchers have tried to model the properties from different perspectives. Using the microscopic perspective, researchers have modeled MSM alloys’ behavior by the variants’ structure and thermodynamic free energy. Thermodynamics of the mechanical and magnetic properties was modelled by Ullakko and Likhachev [12]. O’Handley et al. [13] have discussed the magnetic-field-induced strains of MSM alloys through the relationship between magnetization and twin-boundary motion by minimizing its free energy, which includes Zeeman energy, magnetic anisotropy energy, internal elastic energy and external stress. Hirsinger and Lexcellent [14] introduced a phenomenological model about the variants’ rearrangement through thermodynamics with internal variables, which contains chemical energy, mechanical energy, magnetic and thermal energy. Gauthier et al. [15] combined thermodynamics with Lagrangian formalism and its Hamiltonian extension as a dynamical model for an MSM alloy-based actuator. Tan and Elahinia [16] described the dynamic behavior of a MSM actuator by combining its constitutive model, the reorientation kinetics, the kinematic model, and the dynamic model of the actuator. In these kinds of models, a complete understanding of the material structure and energy flow are necessary. However, to consider all of the influential factors increases the complexity of the model.

Researchers have used a mathematical model to describe the MSM alloys’ hysteresis phenomenon from the macroscopic perspective, which means the MSM alloys are regarded as a black box and the consideration is only its input as source signals and output as strain. The Preisach model is a method used to describe hysteresis and it is suitable for those smart materials possessing this property, such as piezoelectric, SMA, MSM alloys, etc. [17,18,19,20,21]. Inverting the hysteresis models can be used to compensate for tracking control. The MSM actuator is described as a Preisach-like Krasnosel’skii–Pokrovskii model, combining the inverse model as compensation and adaptive control for positioning control by Riccardi et al. [22,23]. In addition to the Preisach model, Sadeghzadeh et al. [24] used phase shift hysteresis compensation to describe the MSM actuator and combined with PID control and gain scheduling for position controller. Zhou et al. [25,26,27] used an inverse Prandtl-Ishlinskii model to eliminate the influence of the hysteresis non-linearity and a PID neural network as hybrid control for the MSM actuator.

In previous studies, fuzzy sliding mode control (FSMC) had been used for MSM actuators to achieve a model-free and robust controller [28], and the modified fuzzy sliding mode control (MFSMC) is proposed by adding an additional integrator in FSMC to prevent the control signal from approaching zero and track the desired position in the steady state [10]. The purpose of this paper is follow-up research based on previous studies. There are some improvements in this paper. First, the test rig is an advanced design, so that the MSM actuator is suitable to work for the purpose of a tracking in the order of micrometers. Second, to enhance tracking ability, the accuracy of tracking error was improved through our novel controller.

In the following sections, firstly, the MSM actuator testbed and the main components are introduced. Section 3 adopts the Preisach model with verified data from experiments to simulate the response of a MSM actuator, and then the inverse Preisach model by numerical recursive implementation in discrete time is applied as hysteresis compensator for the MSM actuator. Section 4 explains the concept of fuzzy sliding mode control. Therefore, the inverse Preisach model, as a position prediction, is a hysteresis compensator and path controller MFSMC is integrated in this paper. Section 5 shows the results of experiments, including results of using inverse Preisach solely, the results of using MFSMC solely, and the results of combining both controllers. Finally, Section 6 is presents the conclusions of the paper.

## 2. MSM Actuator Overview

**Figure 2:**
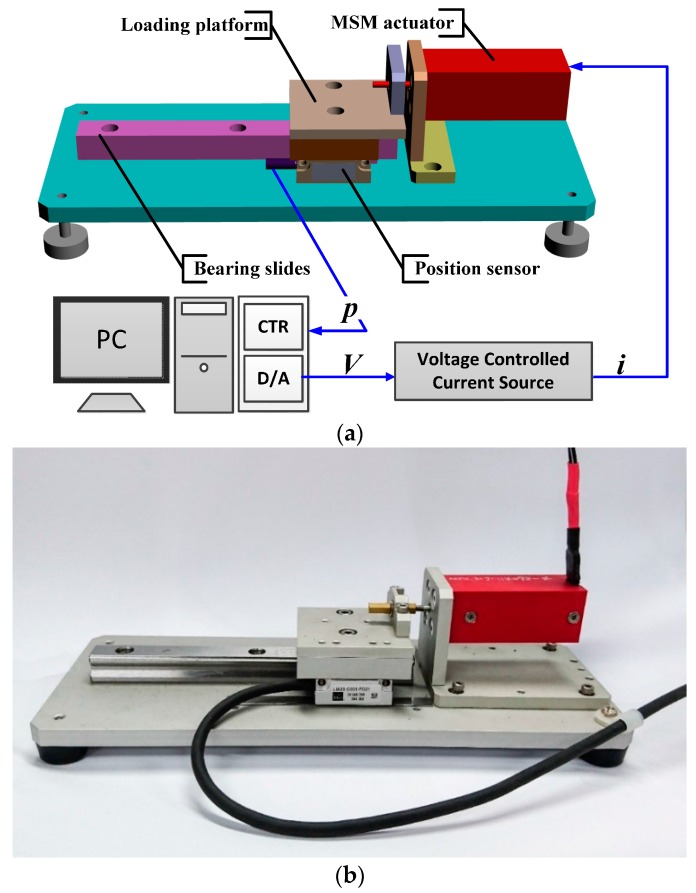
(**a**) Configuration of Test Rig; (**b**) Photo of the MSM actuator.

The MSM actuator experimental setup is shown in Figure 2a, and the photo of the test rig is shown in Figure 2b. The experimental setup is composed of a MSM actuator, bearing slides and a loading platform. The MSM actuator is the product made by AdaptaMat Ltd. (Helsinki, Finland), and its specifications are listed in Table 1. The MSM alloy elongation is induced by the strength of a magnetic field which is passing through it perpendicularly. Increasing the magnetic field elongates MSM alloys, and decreasing the magnetic field compresses MSM alloys by the return spring, which can release the elastic potential energy that was stored from the elongation of MSM alloys. The MSM actuator is driven from a voltage controlled current source that can linearly covert voltages to currents. The position sensor, an optical encoder made by NUMERIK JENA GmbH (Jena, Germany) with resolution of 20 nm, measures the stroke of the MSM actuator. All the signals are sent out and fed back to a PC-based controller via a multifunction DAQ card, made by National Instruments (Austin, TX, USA). The sampling frequency is set as 1000 Hz.

**Figure 3:**
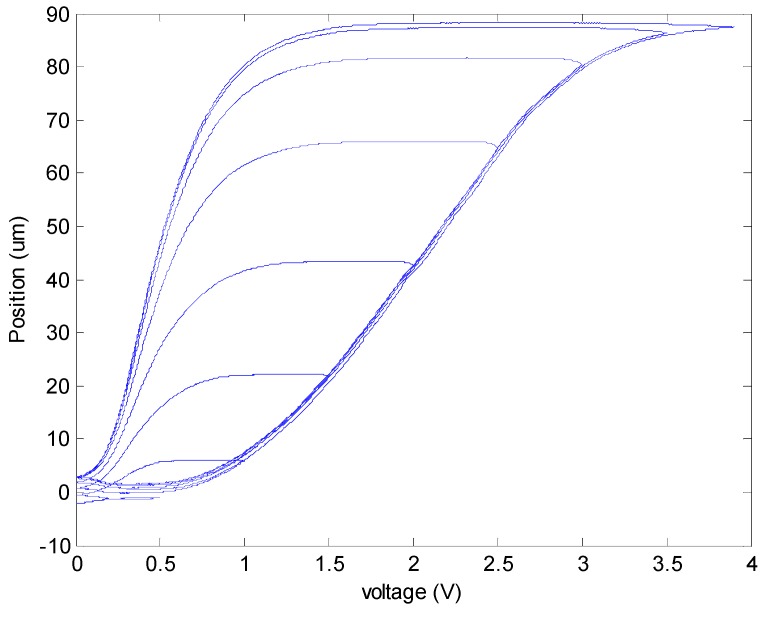
Hysteresis phenomenon of the MSM actuator. Triangle wave voltages range from 0 V to 4 V. After each experiment, subtract 0.5 V each time from the previous voltage until the highest voltage reaches 0.5 V.

**Figure 4:**
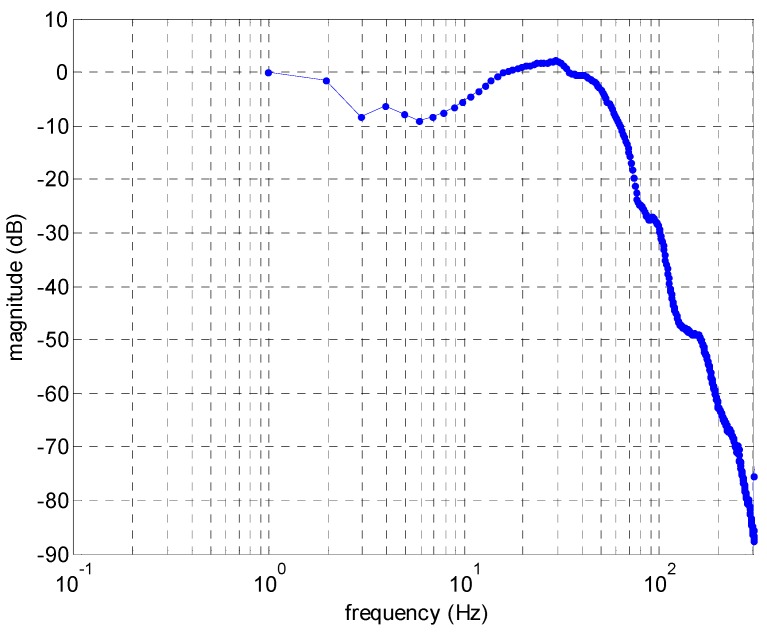
Frequency response of the MSM actuator.

The hysteresis phenomenon of the MSM actuator is shown in Figure 3. Triangular waves with 2 s of rising time and 2 s of falling time are used to drive the MSM actuator. Through the whole experiment, the highest voltage for the MSM actuator is 4 V, which equivalently means that the MSM actuator is driven by a 4 A current. To test hysteresis, the highest voltage of triangle wave was lowered 0.5 V each time until the highest voltage reaches 0.5 V. In the figure it is shown that the longest position is around 88 μm. Frequency response had also been tested as shown in Figure 4. Its frequency response is up to more than 200 Hz, and the largest displacement sits at around 30 Hz.

## 3. Model of Hysteresis and Its Compensator

In the previous section, the hysteresis phenomenon is observed through experiments. Therefore, modeling the hysteresis helps to predict the output position of the MSM actuator. The Preisach model is used in this paper for modeling the MSM actuator. Once the model is constructed, we can use the inverse model to predict the input voltage for tracking specific reference.

### 3.1. The Preisach Model for the MSM Actuator

The Preisach model can be formulated as follows [29]:

(1) $$f(t)=\iint_{\alpha \geq \beta }\mu (\alpha ,\beta )\gamma_{\alpha \beta }[u(t)]d\alpha d\beta$$

where f(t) is the elongation of the MSM actuator; μ(α,β) is a weighting function in the Preisach model; $\gamma_{\alpha \beta }$ is the hysteresis operator; u(t) is the input signal; $\gamma_{\alpha \beta }$ is a function of u(t), and its trigger values are set by α and β.

**Figure 5:**
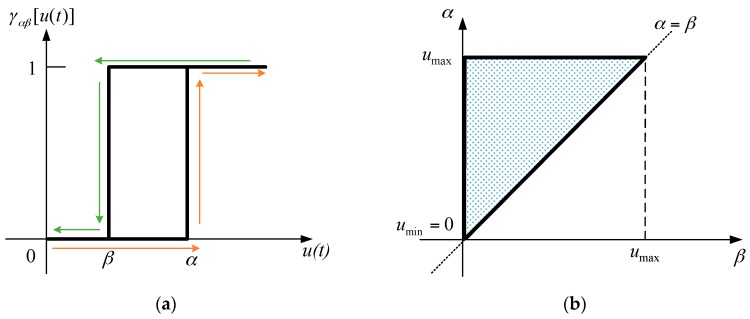
(**a**) Hysteresis operator; (**b**) The Preisach plane.

In the Preisach model, α has to be greater or equal to β. The relationship between $\gamma_{\alpha \beta }$ and u(t) is shown in Figure 5a. As u(t) is monotonically increasing that is shown in orange line, $\gamma_{\alpha \beta }$ is zero until u(t) exceeds α, then $\gamma_{\alpha \beta }$ will switch directly to one afterwards. On the contrary, as u(t) is monotonically decreasing that is shown in green lines, $\gamma_{\alpha \beta }$ will change to zero from one afterwards when u(t) is under β.

The Preisach model in Equation (1) integrates multiple hysteresis operators. All the hysteresis operators can be demonstrated on the Preisach plane as shown in Figure 5b. $u_{max}$ indicates the maximum input value for the MSM actuator, and $u_{min}$ is zero voltage. The *x*-axis represents all the values of β and the *y*-axis represents the values of α. As the definition in Equation (1), α is always equal or larger than β. Therefore, the working area is limited within the upper right triangle as shown in Figure 5b.

In this model, hysteresis is not only influenced by local extrema, which means it is not only related to the current input signal, but it is also influenced by the past position. Hence, it is important to know that saving past extrema is necessary in using the Preisach model.

**Figure 6:**
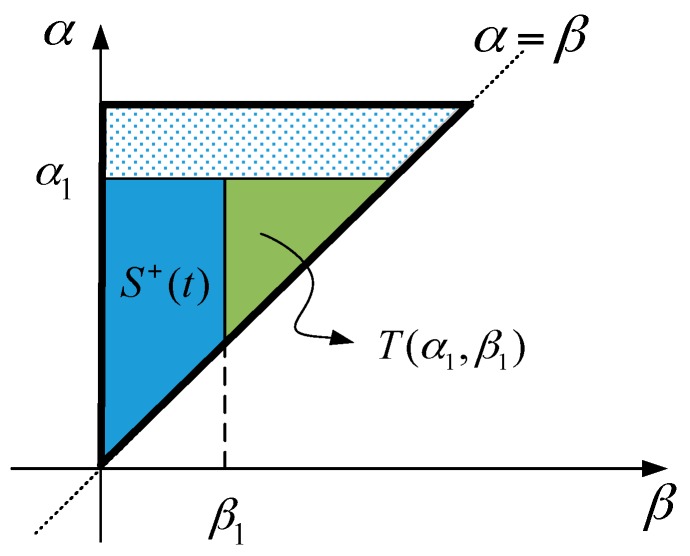
The explanation of area $T\left(\alpha_{1},\;\beta_{1}\right)$ on the Preisach plane.

Next, to implement the Preisach model, a new function *F*(*α*_1_,*β*_1_) is defined as:

(2) $$F(\alpha_{1},\beta_{1})=f_{\alpha_{1}}-f_{\alpha_{1}\beta_{1}}=\iint_{T(\alpha_{1},\beta_{1})}\mu (\alpha ,\beta )d\alpha d\beta$$

where $f_{\alpha_{1}}$ is the output as the input signal increases to $\alpha_{1};$ $f_{\alpha_{1}\beta_{1}}$ is the output as the input signal first reaches $\alpha_{1}$ and then decreases to $\beta_{1}$, and $T\left(\alpha_{1},\;\beta_{1}\right)$ indicates the area between $f_{\alpha_{1}}$ and $f_{\alpha_{1}\beta_{1}}$ on the Preisach plane, as shown in Figure 6.

We derive rising and falling processes of input signals with similar analytically discrete methods. It is now able to calculate the output of the MSM actuator by the following two equations, as the time for input is increasing, Equation (3) is used; as the time for input is decreasing, Equation (4) is used.

(3) $$f(t)=\left\{\sum_{k=1}^{n-1}F(M_{k},m_{k-1})-F(M_{k},m_{k})\right\}+F(u(t),m_{n-1})$$

(4) $$f(t)=\left\{\sum_{k=1}^{n-1}F(M_{k},m_{k-1})-F(M_{k},m_{k})\right\}+F(M_{n},m_{n-1})-F\left(M_{n},u(t)\right)$$

**Figure 7:**
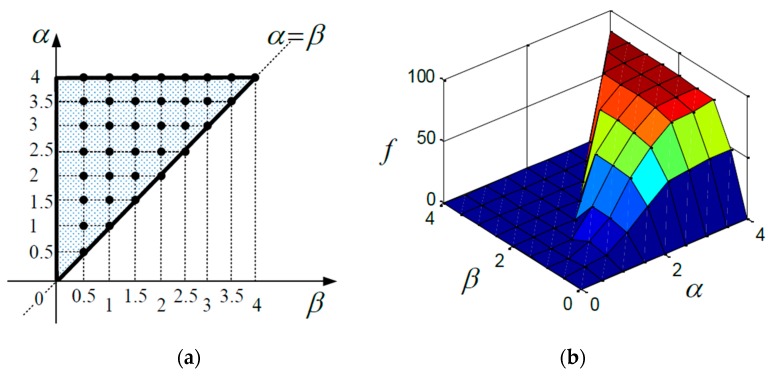
Experimental data recorded for the numerical calculation (**a**) Set points within the working area for data capture; (**b**) the output value for each point.

In order to implement the numerical calculation, it is necessary to record the experimental data. The experimental data are saved for each voltage value. The voltage steps up from 0 V to 4 V in 0.5 V steps. There are in total 36 points on the Preisach plane in Figure 7a, and the experimental values are shown in Figure 7b. Hence, all the points on the Preisach plane can be derived as Equation (5):

(5) $$f_{\alpha \beta }={c_{0}}^{\alpha \beta }+{c_{1}}^{\alpha \beta }\alpha +{c_{2}}^{\alpha \beta }\beta +{c_{3}}^{\alpha \beta }\alpha \beta$$

where $f_{\alpha \beta }$ is the output at (α,β). For calculating the parameters ${c_{0}}^{\alpha \beta }$, ${c_{1}}^{\alpha \beta }$, ${c_{2}}^{\alpha \beta }$ and ${c_{3}}^{\alpha \beta }$, the points on the Preisach plane which are lying within any of squares, the bilinear spline interpolation is used; the points on the Preisach plane which are lying within any of triangles, ${c_{3}}^{\alpha \beta }$ is set to zero and a linear interpolation is used.

### 3.2. The Inverse Preisach Model

In the previous section, the Preisach model is used to predict the MSM actuator’s output for a given input. In this part, the inverse Preisach model is discussed for path tracking control so that the input signals of the MSM actuator can be derived for a giving reference position. Let $f_{r}$ be the desired trajectory, which is linear to the input signal as shown in Equation (6). The compensation signals can be calculated by the inverse Preisach model, and then inputted to the system for eliminating the hysteresis phenomena:

(6) $$f_{r}=A+Bu\left(t\right)$$

**Figure 8:**
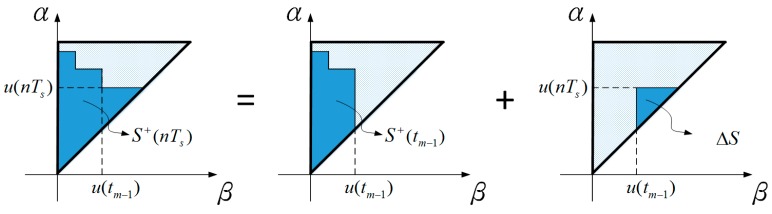
The relationship between input signals and the processing of the Preisach plane.

Since the PC-based computation is giving signal in discrete time with a specific sampling time $T_{s}$, it’s necessary to transfer the Preisach model into a recursive discrete-time model. Equation (7) stands for increasing input, and Figure 8 shows the corresponding area of the symbol.

(7) $$\begin{array}{cc} f\left(nT_{s}\right) & =\iint_{S^{+}\left(nT_{s}\right)}\mu \left(\alpha ,\beta \right)d\alpha d\beta \\ & =\iint_{S^{+}\left(t_{m-1}\right)}\mu \left(\alpha ,\beta \right)d\alpha d\beta +\iint_{\Delta S}\mu \left(\alpha ,\beta \right)d\alpha d\beta ,\text{ for }u\left(nT_{s}\right)>u\left((n-1)T_{s}\right)\\ & =f\left(t_{m-1}\right)+\iint_{\Delta S}\mu \left(\alpha ,\beta \right)d\alpha d\beta \\ & =f\left(t_{m-1}\right)+f_{u\left(nT_{s}\right)}-f_{u\left(nT_{s}\right)u\left(t_{m-1}\right)} \end{array}$$

where $nT_{s}$ indicates the present discrete time, $\left(n-1\right)T_{s}$ is the previous sampling time. $u\left(nT_{s}\right)$ is the last extrema input. $t_{m-1}$ is the time of the previous extrema input, and $f\left(t_{m-1}\right)$ is the output for the previous extrema input. If the input decreases, it can be derived by the same method and shown in Equation (8).

(8) $$f\left(nT_{s}\right)=f\left(t_{m-1}\right)+f_{u\left(t_{m-1}\right)u\left(nT_{s}\right)}-f_{u\left(t_{m-1}\right)}\text{ for }u\left(nT_{s}\right)<u\left((n-1)T_{s}\right)$$

Therefore, combing Equation (5) with the experimental data and Equations (7) and (8), we can get the compensational signals as shown in Equation (9) for increasing signals and Equation (10) for decreasing signals:

For $u\left(nT_{s}\right)>u\left((n-1)T_{s}\right),$

(9) $$v\left(nT_{s}\right)=\frac{A+Bu\left(nT_{s}\right)-f^{p}\left(t_{m-1}\right)-\left(c_{0}^{v\left(nT_{s}\right)v\left(nT_{s}\right)}-c_{0}^{v\left(nT_{s}\right)v\left(t_{m-1}\right)}-c_{2}^{v\left(nT_{s}\right)v\left(t_{m-1}\right)}v\left(t_{m-1}\right)\right)}{c_{1}^{v\left(nT_{s}\right)v\left(nT_{s}\right)}+c_{2}^{v\left(nT_{s}\right)v\left(nT_{s}\right)}-c_{1}^{v\left(nT_{s}\right)v\left(t_{m-1}\right)}-c_{3}^{v\left(nT_{s}\right)v\left(t_{m-1}\right)}v\left(t_{m-1}\right)}$$

For $u\left(nT_{s}\right)<u\left((n-1)T_{s}\right),$

(10) $$v\left(nT_{s}\right)=\frac{\left(\begin{array}{l} A+Bu\left(nT_{s}\right)-f^{p}\left(t_{m-1}\right)-{c_{0}}^{v\left(t_{m-1}\right)v\left(nT_{s}\right)}-{c_{1}}^{v\left(t_{m-1}\right)v\left(nT_{s}\right)}v\left(t_{m-1}\right)+\ldots \\ {c_{0}}^{v\left(t_{m-1}\right)v\left(t_{m-1}\right)}+{c_{1}}^{v\left(t_{m-1}\right)v\left(t_{m-1}\right)}v\left(t_{m-1}\right)+{c_{2}}^{v\left(t_{m-1}\right)v\left(t_{m-1}\right)}v\left(t_{m-1}\right)+{c_{3}}^{v\left(t_{m-1}\right)v\left(t_{m-1}\right)}v{\left(t_{m-1}\right)}^{2} \end{array}\right)}{{c_{2}}^{v\left(t_{m-1}\right)v\left(nT_{s}\right)}+{c_{3}}^{v\left(t_{m-1}\right)v\left(nT_{s}\right)}v\left(t_{m-1}\right)}$$

## 4. Controller Design

### 4.1. Concept of Modified Fuzzy Sliding Mode Control

**Figure 9:**
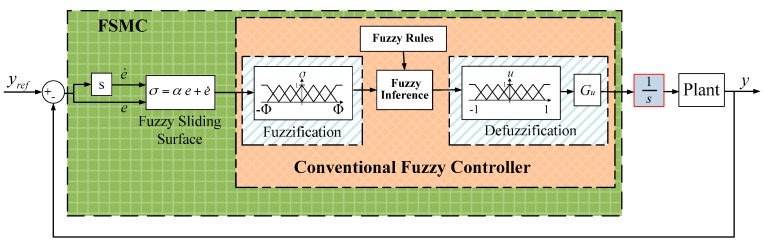
Block diagram of MFSMC system.

Figure 9 depicts the block diagram of the modified fuzzy sliding mode control system. Fuzzy logic is used for fuzzy controller, which includes fuzzification, a fuzzy rule base, fuzzy inference, and defuzzification. However, the fuzzy inference rules will increase and the membership functions will be complicated if the fuzzy control system needs to consider both control e and error rate $\dot{e}$. For instance, to construct seven rules of error e and seven rules of error rate $\dot{e}$ for fuzzy logic, 49 rules are needed. It is complex and memory-intensive for real-time computation. Fuzzy sliding mode control (FSMC) combines the advantages from fuzzy control and sliding mode control. The FSMC system uses a fuzzy sliding surface $\sigma =\alpha e+\dot{e}$, which is a linear combination of error rate $\dot{e}$ and error e, so that the dimensions of the input space and the number of fuzzy inference rules can be reduced. Due to the fuzzy rules, the output u approaches zero if σ is close to zero. In modified fuzzy sliding mode control (MFSMC), an integrator is inserted after output from the fuzzy logic control, so that the MSM actuator can keep its position to the desired position as the error approaches zero. Lyapunov’s theory is used to guarantee the stability and the bound of the tracking error of the system.

### 4.2. Designing MFSMC for Path Controller

A non-linear system can be described as follows [10]:

(11) $$x^{\left(n\right)}(t)=f(x,t)+d(t)+u,\;x^{\left(n\right)}=\frac{d^{n}x}{dt^{n}}$$

where $x\left(t\right)={\left[x,\dot{x},\ldots ,x^{\left(n-1\right)}\right]}^{T}$ is the state vector; f(x,t) is a non-linear function with a bound of F(x,t); d(t) is a disturbance bounded by D(t) and *u* is the control input. The tracking error of the state vector is defined as:

(12) $$e(t)=x(t)-x_{d}(t)={\left[e,\dot{e},\ldots ,e^{\left(n-1\right)}\right]}^{T}$$

where $x_{d}\left(t\right)$ is the desired output. 

The fuzzy sliding surface is defined as follows:

(13) $$\sigma (x,t)={\left(\frac{d}{dt}+\alpha \right)}^{n-1}\;e=0$$

where α is a positive constant [30]. To simply the system, set the fuzzy sliding surface as the second order system as:

(14) $$\sigma =(\dot{e}+\alpha \;e)=ZERO$$

where α is the slope of the fuzzy sliding surface σ=ZERO.

The fuzzy sliding surface σ is divided by Φ before fuzzification, and Φ is the boundary layer of the fuzzy sliding surface σ. M(σ)={NB,NM,NS,ZR,PS,PM,PB} is the membership function set of the fuzzy sliding surface σ, where NB, NM, NS, ZR, PS, PM and PB are negative big, negative medium, negative small, zero, positive small, positive medium and positive big. M(u)={NB,NM,NS,ZR,PS,PM,PB} is the membership function set of control input $u_{f}$.

In the conventional fuzzy control, control error e and error rate $\dot{e}$ need to build a 7×7 fuzzy rules if each variable is divided by seven fuzzy rules. Only seven fuzzy rules are necessary for fuzzy sliding mode control. The simplified fuzzy rule base is demonstrated in Table 2.

The Mamdani method is used for fuzzy inference and the center-of-area defuzzifier is used for defuzzification:

(15) $$u_{f}=G_{u}(x,t)\cdot \frac{\int \mu (u)\cdot u\cdot du}{\int \mu (u)\cdot du}$$

where µ(u) indicates the membership function of u.

However, the control signal of FSMC will approach zero if the position control error approaches zero in the steady state. In addition, the MSM actuator will become zero position output. MFSMC is proposed to integrate additional integrator to FSMC [10]. Therefore, the MSM actuator can keep the desired position, since the control signal can be preserved in a steady output value as the position error approaching zero in the steady state.

### 4.3. Novel Controller Design by Combining Path Controller and Hysteresis Compensator

**Figure 10:**
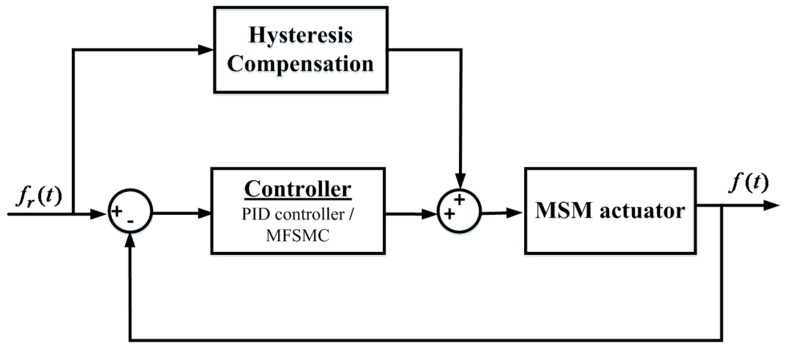
Block diagram of novel controller design by combining path controller and the hysteresis compensator.

The control strategy combines the inverse Preisach model as hysteresis compensator and path controller, which use PID controller or MFSMC. According to Section 3, the MSM actuator’s hysteresis phenomenon can be linearized by the hysteresis compensator. However, the hysteresis compensator is a feedforward control, which is not an error based control. Negative feedback errors can adjust tracking through path controller, i.e., PID controller and MFSMC in the experiments. Path controller’s performance had been tested in the tracking path [10]. However, the obvious delay of the MSM actuator was induced during the change of direction by the friction of the system. To enable a good path tracking in this state is difficult for path controller. This research tackles this problem with the novel controller, which is able to reduce the influence of friction. To verify the performance of MFSMC, PID controller is used for comparison. The control block diagram of the novel controller with the combined path controller and the hysteresis compensator is shown in Figure 10.

## 5. Experiments

In this section, path tracking control of the MSM actuator is implemented. First, we test the performance of the forward controller, i.e., the hysteresis compensator. Then, path controllers, such as PID control and MFSMC, are also tested separately. Finally, the novel controller combined with the hysteresis compensator and path controller is implemented.

### 5.1. Hysteresis Compensator by the Inverse Preisach Model

**Figure 11:**
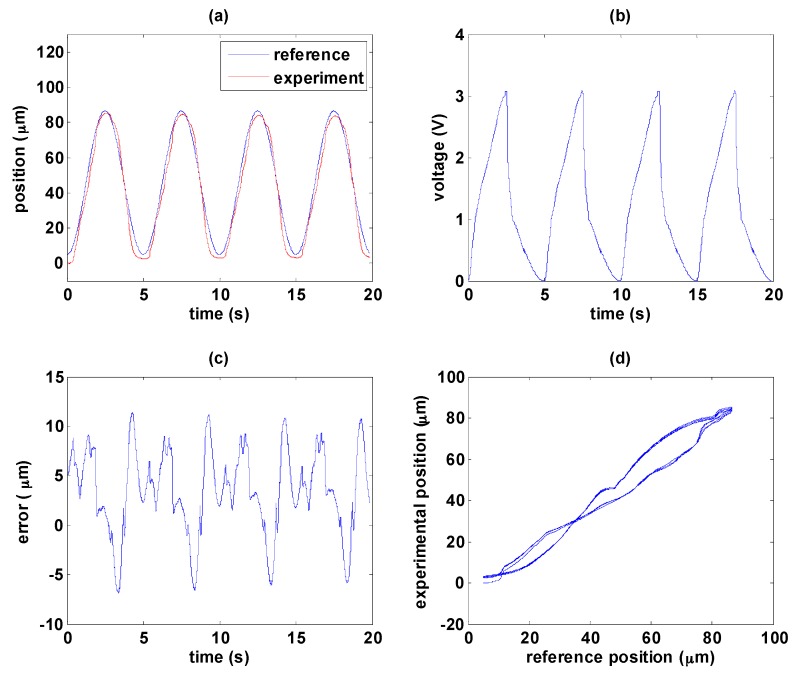
Path tracking of sinusoidal wave by the hysteresis compensator: (**a**) path tracking responses; (**b**) control signals; (**c**) control errors; (**d**) linear relationship between reference and experimental positions.

In the following experiments, the path tracking reference is a sinusoidal wave with amplitude of 81.5 µm and frequency of 0.2 Hz. As mentioned previously the MSM actuator is sensitive to disturbance so that the temperature is kept around 35 °C during the experiment. The experimental results with only the hysteresis compensator are shown in Figure 11. Figure 11a shows the path tracking control response. Nonlinearity of the MSM actuator can be observed clearly in Figure 11b, and rapid falling of input signal is required to return the position back to its original state with good response. The control error, caused mainly by friction force and temperature variation, can be maintained within 10 µm as shown in Figure 11c, especially by the change of moving direction. Besides, temperature changes during the experiment should be another reason causing fluctuations. The magnetic circuit is driven by electric current such that the MSM alloys tend to have temperature change. However, the hysteresis compensator can linearize the MSM actuator with limited effect, as shown in Figure 11d.

### 5.2. Path Tracking Control without the Hysteresis Compensator

**Figure 12:**
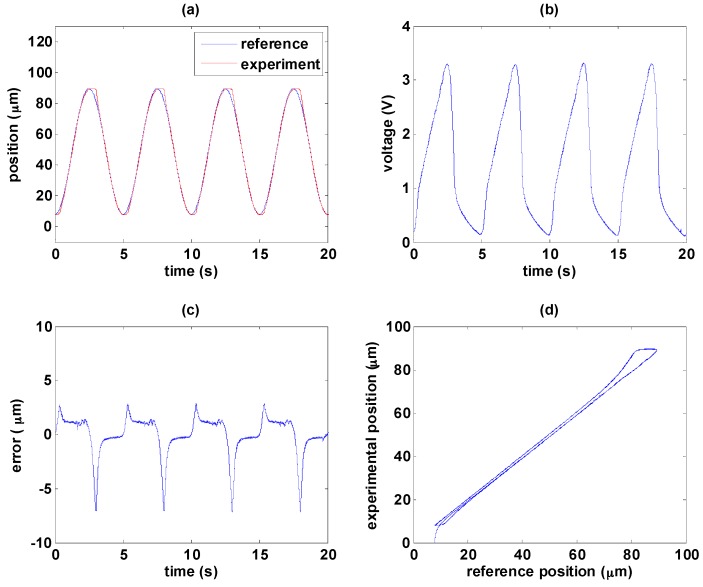
Path tracking of sinusoidal wave with PID controller: (**a**) path tracking responses; (**b**) control signals; (**c**) control errors; (**d**) linear relationship between reference and experimental positions.

**Figure 13:**
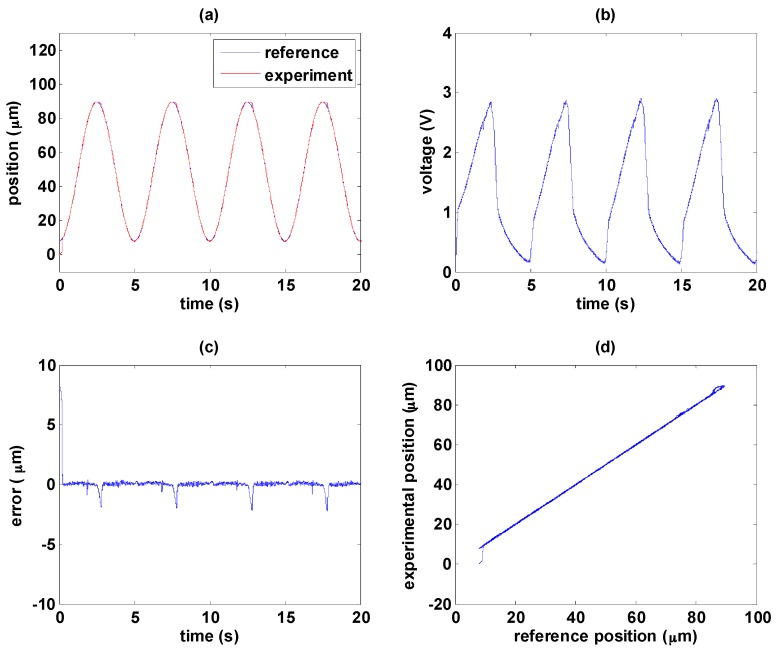
Path tracking of sinusoidal wave with MFSMC: (**a**) path tracking responses; (**b**) control signals; (**c**) control errors; (**d**) linear relationship between reference and experimental positions.

This section shows the experimental results of the path tracking control with two different path controllers, i.e., PID controller and MFSMC. The experimental results of PID controller and MFSMC are shown in Figure 12 and Figure 13, respectively. Friction during direction change causes the maximum error, especially in the transition from elongation to contraction. As the MSM actuator has serious hysteresis, the PID controller cannot tackle this nonlinearity well. It can be noticed in Figure 12a that hysteresis results in delay of the PID controller during the transition of the MSM actuator from elongation to contraction which is due to the falling rates of input signals are not fast enough in PID controller. MFSMC has better performance at the transition period as shown in Figure 13a, which is attributed to the fast switching in the sliding surface of MFSMC. The maximum error at the transition is around 2 µm, and during the period of elongation and contraction, the error can be maintained under 500 nm as shown in Figure 13c.

In comparison with the maximum error by using PID controller of about 7 μm in Figure 12c, it is evident that the MFSMC has better tracking performance than the PID control. Besides, compared the experimental results with that of the hysteresis compensator in Section 5.1, the hysteresis compensator serves as forward control so that it cannot receive the errors to modify the tracking performance. Therefore, the tracking accuracy of the hysteresis compensator in Section 5.1 is limited.

### 5.3. Novel Controller by Combination of Path Controller with the Hysteresis Compensator

**Figure 14:**
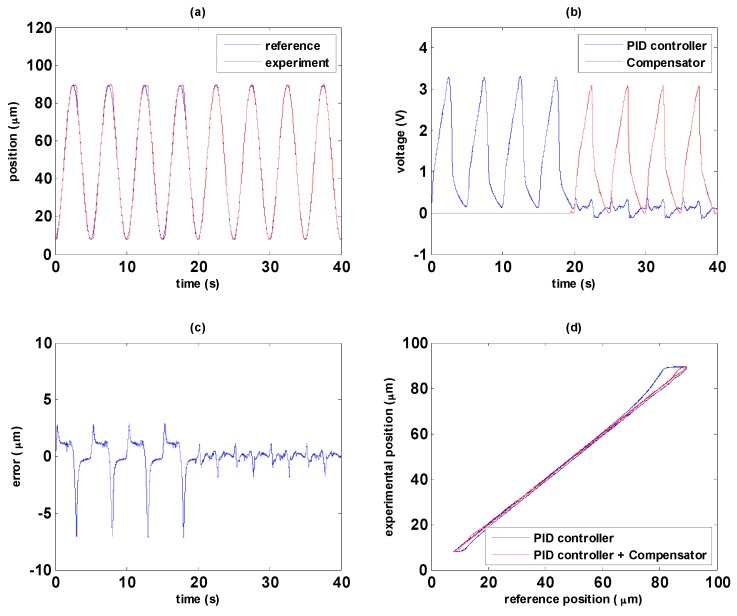
Comparison of PID path control with and without the hysteresis compensator: (**a**) path tracking responses; (**b**) signals of PID controller and the hysteresis compensator; (**c**) control errors; (**d**) linear relationship between reference and experimental positions.

**Figure 15:**
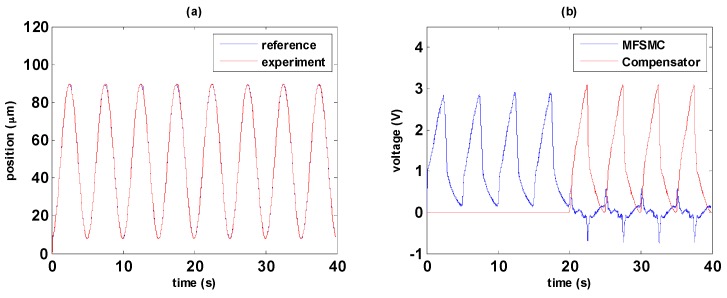

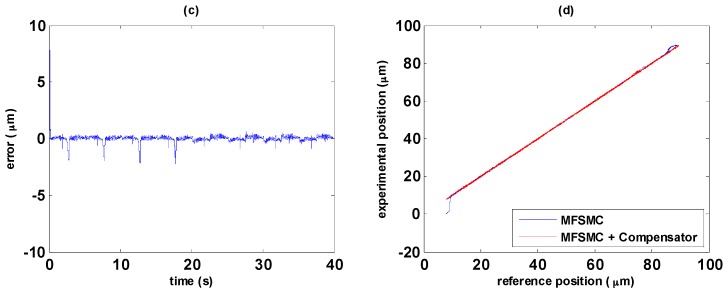
Comparison of MFSMC path control with and without the hysteresis compensator: (**a**) path tracking responses; (**b**) signals of PID controller and the hysteresis compensator; (**c**) control errors; (**d**) linear relationship between reference and experimental positions.

In this section, control strategy experiments are performed by combining the hysteresis compensator and path controller. In order to analyze the path tracking results in an experiment, path controller is applied first, and then the hysteresis compensator is added, i.e., path controller with the hysteresis compensator, at the time point of 20 s. The experimental results of PID path controller with the hysteresis compensator are shown in Figure 14; the experimental results of MFSMC path controller with the hysteresis compensator are shown in Figure 15.

The path tracking responses are shown in Figure 14a and Figure 15a. In the first 20 s, only path controller works for tracking control, and the hysteresis compensator is added after 20 s for achieving the combination of path control and the hysteresis compensator. The hysteresis compensator becomes the major control signals, and path controllers turn out to be the modifying signal for correcting control signals as shown in Figure 14b and Figure 15b. In both experimental results, it can be seen that path controller combining with the hysteresis compensator provides better control performance. In Figure 14c, it can be seen that total errors can be reduced from 7 µm to 2 µm through the PID path controller with the hysteresis compensator. Furthermore, the total errors can be reduced from 2 µm to 500 nm through the MFSMC path controller with the hysteresis compensator as shown in Figure 15c. The linear relationship between the reference and experimental position can be improved obviously as shown in Figure 14d and Figure 15d by the combination of path controller with the hysteresis compensator.

**Figure 16:**
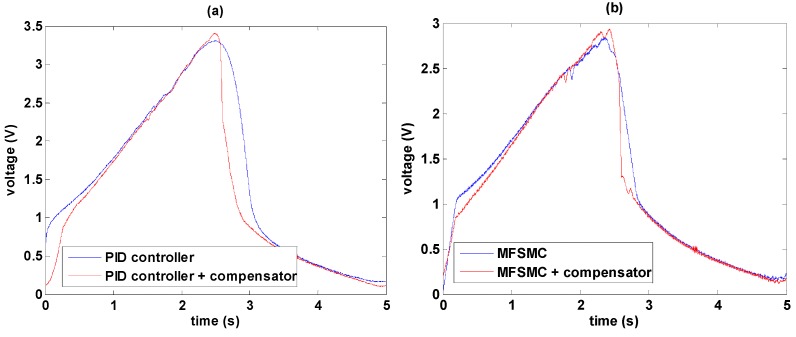
Control signal comparisons between using path controller solely and using path controller with the hysteresis compensator: (**a**) Using PID path controller solely and using PID path controller with the hysteresis compensator; (**b**) Using MFSMC path controller solely and using MFSMC path controller with the hysteresis compensator.

Figure 16 shows the comparisons of control signals with and without the hysteresis compensator. According to Figure 14b, the control signals from 0 to 5 s as PID control and that from 20 to 25 s as PID path control with the hysteresis compensator are selected for comparison and shown in Figure 16a. Similarly, consistent with Figure 15b the control signals from 0 to 5 s as MFSMC path control and that from 20 to 25 s as MFSMC path control with the hysteresis compensator are compared in Figure 16b.

It is obvious that the control signal is especially modified during the changing of direction. Due to the friction, only using path controller cannot adjust the output fast enough so as to cause delay and errors. By combining path controller with the hysteresis compensator, it can be noticed that the signals has fast response to overcome the delay. Moreover, MFSMC with the hysteresis compensator can perform faster response than PID controller with the hysteresis compensator.

### 5.4. Novel Controller in Different Trajectories Control

We have demonstrated the tracking performance with our novel controller. The hysteresis compensator is provided the main input signal for path tracking, and it is able to give the proper response as path changing. In addition, the path controller is able to enhance tracking accuracy. More experiments to verify the performance of the novel controller follow.

#### 5.4.1. Decaying Sinusoidal Trajectory Control

**Figure 17:**
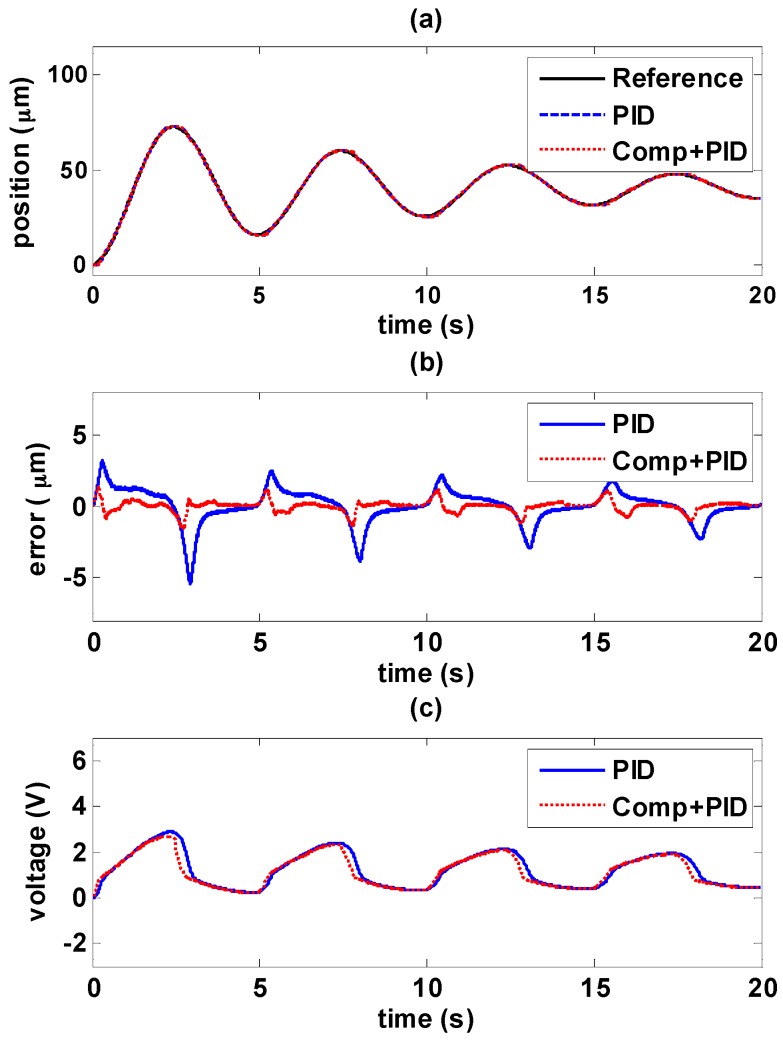
Tracking control comparisons of decaying sinusoidal trajectories with 0.2 Hz frequency between using PID path controller solely and PID path controller with the hysteresis compensator: (**a**) path tracking responses; (**b**) control errors; (**c**) control signals.

**Figure 18:**
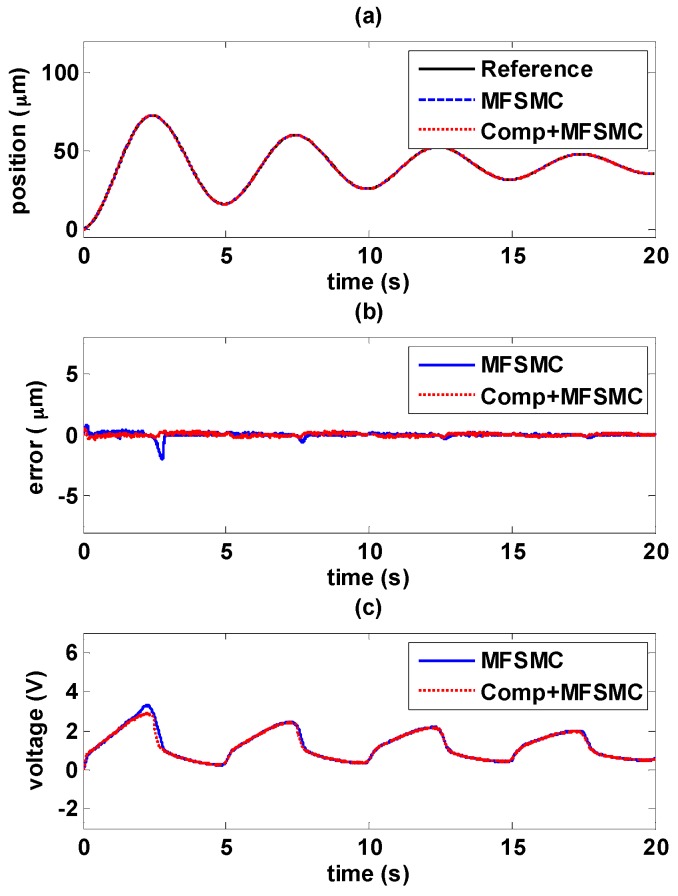
Tracking control comparisons of decaying sinusoidal trajectories with 0.2 Hz frequency between using MFSMC path controller solely and MFSMC path controller with the hysteresis compensator: (**a**) path tracking responses; (**b**) control errors; (**c**) control signals.

The experiments of decaying sinusoidal wave with 0.2 Hz are shown in Figure 17 and Figure 18, and the tracking reference is set as $f_{r}=41e^{-0.1t}\sin\left(0.4\pi t-0.5\pi \right)+82$. It is shown that the tracking performance by using the novel controller is better than by only using path controller in both experiments. The maximum error occurs at the changing of direction of maximum amplitude. As the amplitude decays during the time, the error is also reduced. In Figure 17b, it is shown the maximum error is 5.4 μm with PID controller, and the maximum error is reduced to 1.36 μm by adding the hysteresis compensator. Likewise, in Figure 18b, the maximum error is 1.96 μm with MFSMC, and the total errors can be kept small enough within 450 nm. It can be seen how the input signals is modified by adding the hysteresis compensator.

#### 5.4.2. Sinusoidal Trajectory Control in Higher Frequency

**Figure 19:**
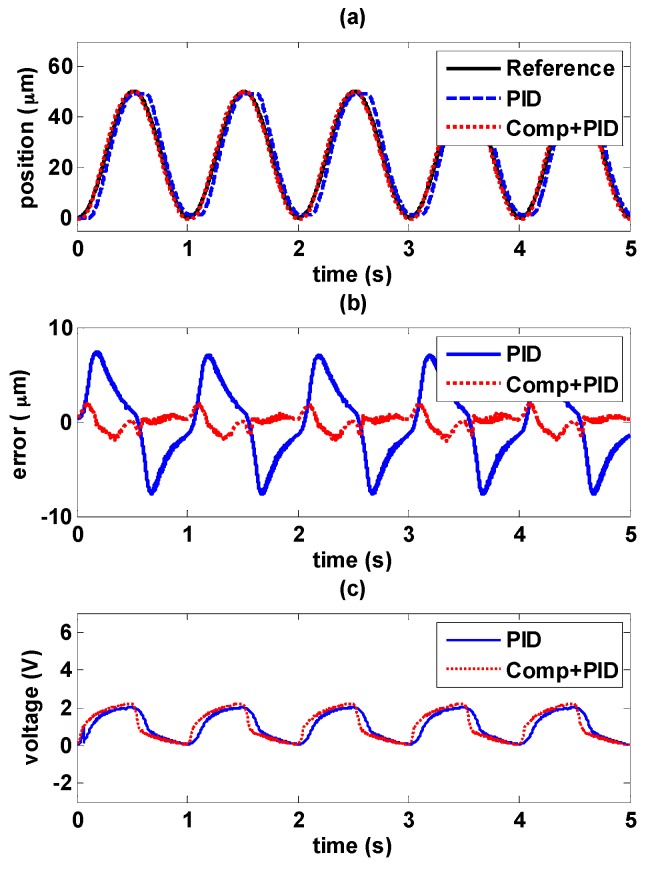
Tracking control comparisons of sinusoidal trajectory with 1 Hz frequency and 50 μm amplitude between using PID path controller solely and PID path controller with the hysteresis compensator: (**a**) path tracking responses; (**b**) control errors; (**c**) control signals.

**Figure 20:**
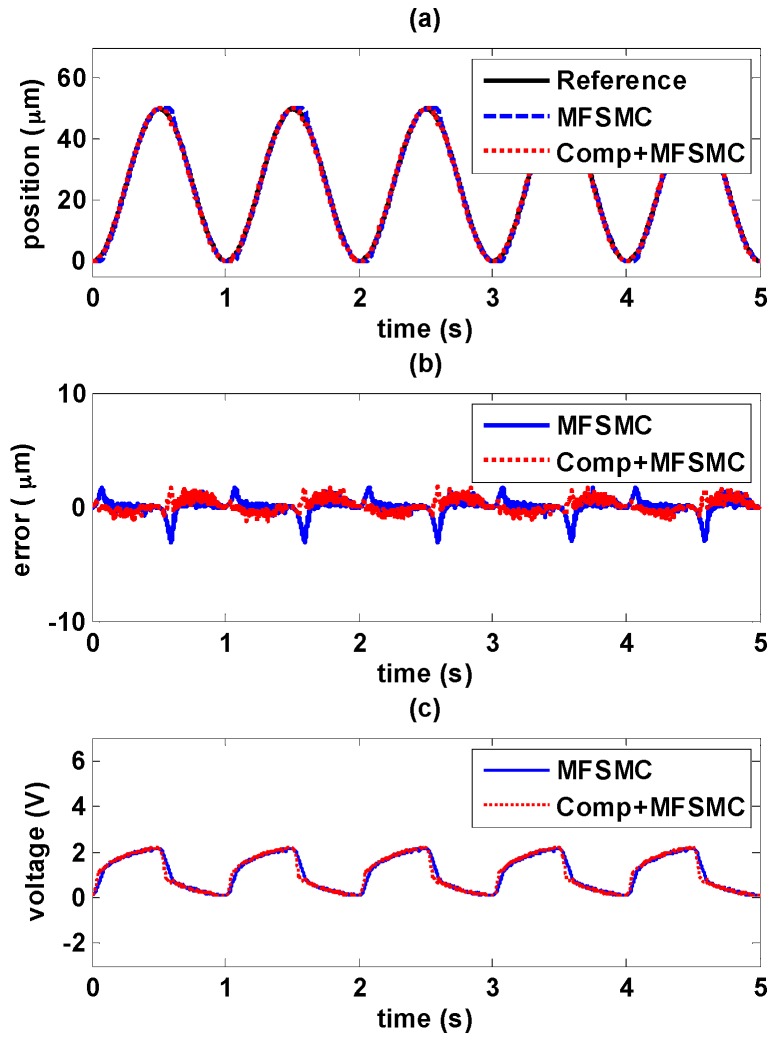
Tracking control comparisons of sinusoidal trajectory with 1 Hz frequency and 50 μm amplitude between using MFSMC path controller solely and MFSMC path controller with the hysteresis compensator: (**a**) path tracking responses; (**b**) control errors; (**c**) control signals.

Sinusoidal wave with the amplitude of 50 μm and higher frequency at 1 Hz is tested and shown in Figure 19 and Figure 20. 

It is evident that higher frequency signals have larger errors. In Figure 19b, the maximum error increases to 7.6 μm with PID controller, and it can be seen that the controller response is much delayed with higher frequency signals as shown in Figure 19c. Adding the hysteresis compensator can improve the tracking performance, the maximum error is able to be reduced to less than 2 μm. Similarly as shown in Figure 20b, 3 μm is the maximum error with MFSMC, and it can be reduced further to less than 1.7 μm by adding the hysteresis compensator.

#### 5.4.3. 5th Order Trajectory Control in Higher Frequency

**Figure 21:**
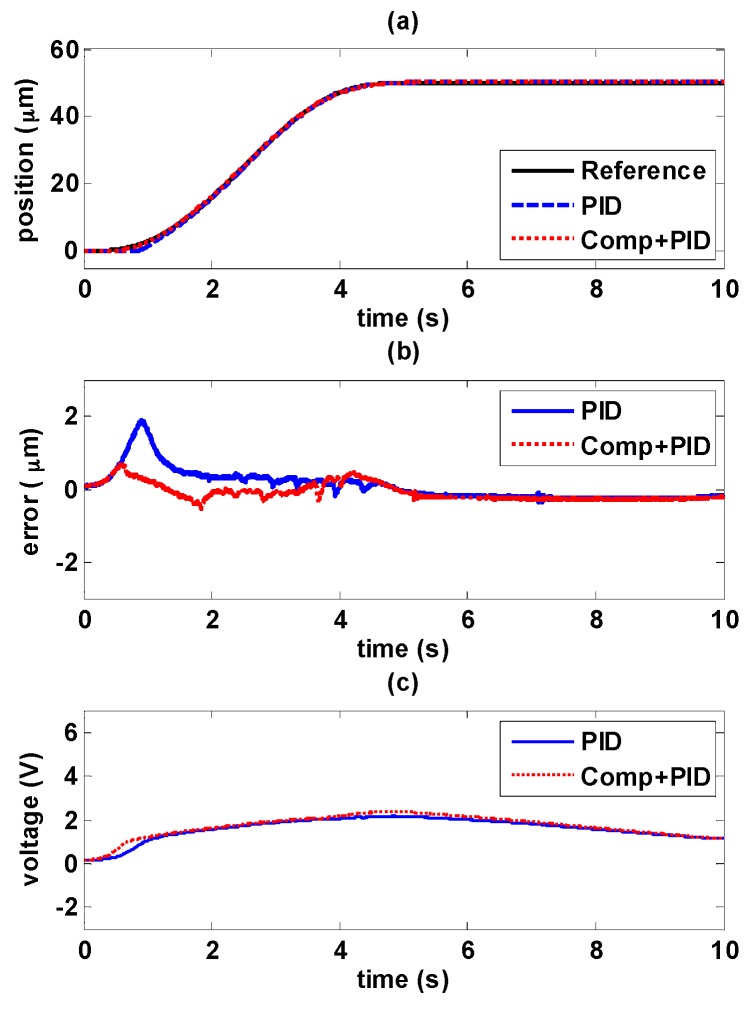
Tracking control comparisons of 5th order trajectory between using PID path controller solely and PID path controller with the hysteresis compensator: (**a**) path tracking responses; (**b**) control errors; (**c**) control signals.

**Figure 22:**
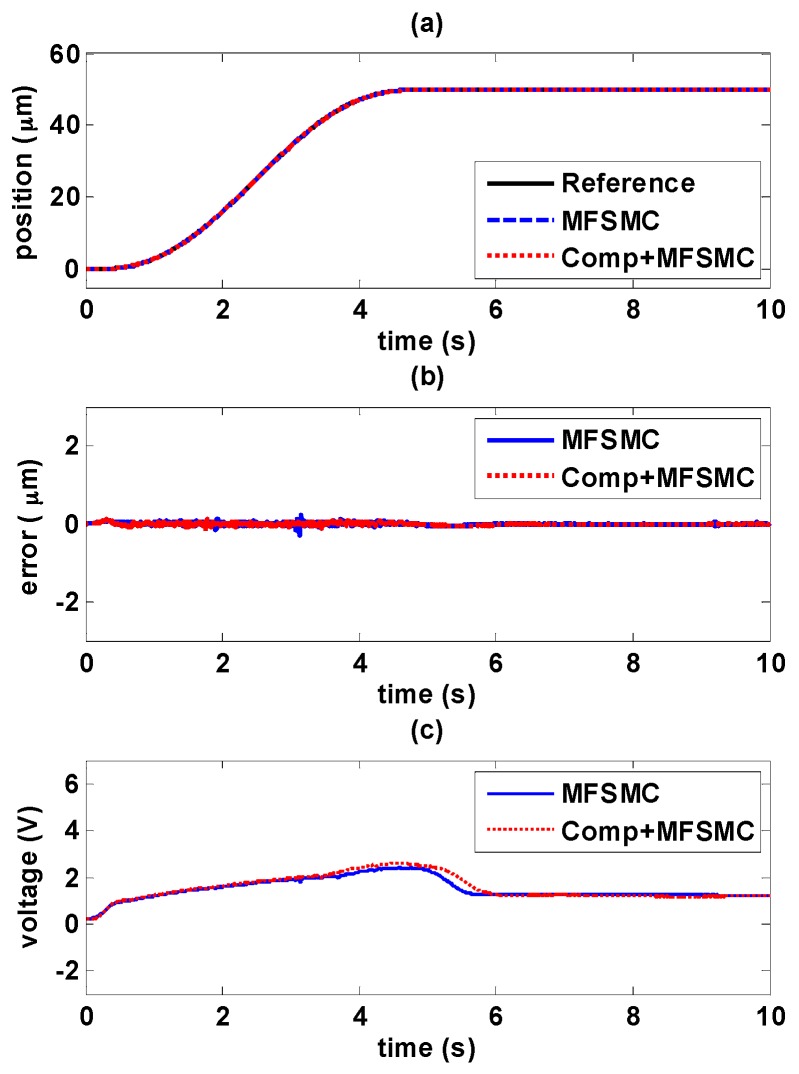
Tracking control comparisons of 5th order trajectory between using MFSMC path controller solely and MFSMC path controller with the hysteresis compensator: (**a**) path tracking responses; (**b**) control errors; (**c**) control signals.

In these experiments, 5th order trajectory is used as a smooth tracking path for the MSM actuator. 5th order trajectory has continuous function in position, velocity and acceleration. Therefore, it is evident that the errors are small in Figure 21 and Figure 22. In Figure 21b, the maximum error is 1.87 μm, which is controlled by PID controller solely, and it has large friction because the speed of the MSM actuator is slow. Besides, maximum error can be reduced to 660 nm by adding the hysteresis compensator, and the steady state error is 280 nm. For MFSMC, the error is much less than PID controller with the hysteresis compensator. It is able to be reduced to less than 280 nm for maximum error, and the steady state error is able to reach to 20 nm, which is the resolution of position sensor. Adding the hysteresis compensator for MFSMC, 134 nm is the maximum error, and the steady state error is able to be kept as small as 20 nm.

## 6. Conclusions

The MSM actuator inherits the advantageous properties of huge strain and fast response like MSM alloys, however, they also exhibit a hysteresis phenomenon. This paper aims to develop a novel controller by combining a path controller with a hysteresis compensator for path tracking control of MSM actuators.

The Preisach model was used to model the hysteresis phenomenon. The hysteresis of the MSM actuator was modeled on a Preisach plane by collecting experimental data. The desired position could be achieved by applying the inverse Preisach model, which was also used as a hysteresis compensator. The novel controller combined the hysteresis compensator and path controller. The path controllers, including PID controller and MFSMC, were implemented for comparison.

Different controller trajectories were tested, including sinusoidal wave, decaying sinusoidal wave, sinusoidal wave in higher frequency and 5th order trajectory. It was shown that the MFSMC path control can perform better than PID controller in the path tracking control experiments. Besides, the novel controller by combining path controllers with the hysteresis compensator was able to improve tracking accuracy. The path tracking control experiments verified that the best performance was the MFSMC path controller with the hysteresis compensator.

## Figures and Tables

**Table 1:** Specifications of the MSM actuator.

Items	Speciafication	Company
Actuating element	5M Ni-Mn-Ga MSM element	AdaptaMat Ltd.
Element size:	20 × 2.5 × 1.0 mm^3^	
Fatigue life	200 million cycles (minimum)	
Rise time	1 ms	
Frequency response	≥250 Hz	
Maximum power dissipation	3.5 W	
Actuator dimensions	25 × 25 × 66 mm^3^	
Weight	160 g	
Data Acquisition Card: output	16-bit analog outputs (833 kS/s)	National Instruments Ltd.
Data Acquisition Card: counters	32-bit counters	
Optical Encoder	Resolution: 20 nm	NUMERIK JENA GmbH

**Table 2:** Fuzzy Rules.

Rules	IF	THEN
$R^{1}$	σ is PB	u is PB
$R^{2}$	σ is PM	u is PM
$R^{3}$	σ is PS	u is PS
$R^{4}$	σ is ZR	u is ZR
$R^{5}$	σ is NS	u is NS
$R^{6}$	σ is NM	u is NM
$R^{7}$	σ is NB	u is NB

## Abbreviations

MSM	Magnetic Shape Memory
FSMC	Fuzzy Sliding Mode Control
MFSMC	Modified Fuzzy Sliding Mode Control
